# Derivation of Local Anesthetic Drugs Used in Dental Practice

**Published:** 1910-10-15

**Authors:** W. H. T. Collins

**Affiliations:** Philadelphia


### DERIVATION OF LOCAL ANESTHETIC DRUGS USED IN DENTAL PRACTICE.

BY W. H. T. COLLINS, D.D.S., PHILADELPHIA.

Cocain is derived from the dried leaves of the erythroxylon coca, a small tree or shrub, a native of the west coast of South Africa. Its original habitat was the mountain regions of Peru, between latitudes seven degrees south and ten degrees north. It may now be found anywhere on the eastern slope of the Andes mountains at an elevation of from 1,500 to 6,000 feet, and from the Straits of Magellan to the Carribbean Sea. Moisture and an even temperature are necessary for its cultivation. There are many varieties of the shrub from which cocain is obtained, but as they differ greatly in appearance, the one from the other, an accurate description of each cannot be given in so short a paper.

The two principal commercial varieties are the huanuco coca and the truxillo coca. The former is a native of Boliva, Huanuco, Brazil, Venezuela and Argentina. The latter is found in the mountains regions of Northern Peru. The wild shrub grows to the height of from twelve to twenty feet, but the cultivated variety is kept trimmed to an average height of about six feet.

The shrub begins to yield in about eighteen months and continues to yield for a half a century. It produces from three crops annually. The first crop is gathered the cut-off twigs at the time the tree is trimmed. At the end of June a smaller crop is gathered, and in October or November the final crop is taken off. It is necessary that the crops be gathered in dry weather, in order that the fresh leaves, when spread out on the drying pavement in layers of two or three inches in thickness, may be sufficiently dried in from six to eight hours. The leaves, when matured, are carefully picked by hand, to avoid breaking or injuring the young buds. They are then dried in the sun and packed in bags holding from twenty-five to one hundred and fifty pounds each.

The plant was used by the Peruvians long before the time of the conquest as a stimulant, to ward off a sense of hunger and to increase their power of endurance when taking long journies. It is estimated that forty million pounds, or ten million dollars' worth, are each year produced in South America at the present time.

The process of extracting the alkaloid, as given by Alfred Nieman, is as follows:

The leaves are exhausted with an 85 per cent. solution of alcohol, acidulated with a 2 per cent. sulphuric acid solution. The resulting tincture is treated with milk of lime and filtered. The filtrate is neutralized with sulphuric acid, and the alcohol removed by evaporation. The syrupy residue is then treated with water to remove the resin, and the cocain precipitated with sodium carbonate. The deposited matter is exhausted with ether, the ether partially removed by distilling, and the remainder allowed to evaporate spontaneously. The cocain is thus obtained in colorless crystals, mixed with a yellow brown matter of unpleasant odor, which is removed by washing with cold alcohol.

A practical objection to cocain is its comparatively insolubility in water. To overcome this it is treated with hydrochloric acid, producing cocain hydrochlorid, a very much more soluble drug, retaining to a marked degree all the useful properties of the alkaloid.

Mr. R. E. Squibbs has devised a method by which the hydrochlorid is obtained more direct.

Coarsely ground coca leaves are repercolated with a 5 per cent. aqueous sulphuric acid solutoin. A dense, slightly acid percolate is obtained.

This percolate is mixed with pure coal oil, and an excess of sodium carbonate. The liberated alkaloid is retained by the coal oil, and is nearly free from coloring matter. The oily solution is mixed with acidulated water, and again precipitated with sodium carbonate in the prescence of ether.

The etherial solution of cocain is treated with dilute hydrochloric acid, fractionally, and the solutions of cocain hydrochlorid are carefully evaporated. The product is in the form of a white, crystaline, granular powder, and is a nearly anhydrous salt.

A two to ten per cent, solution of the official salt of the drug, cocain hydrochloride, may be used for hypodermic injection, but great care must be exercised on account of its toxicity.

The number of cases of poisoning by cocain is very great, and although large doses have been recovered from, very violent symptoms have followed the administration of smaller doses. It is remarkable, also, that a large percentage of these cases have been the result of local application, especially as used in dentistry.

According to the observations of Von Anrep, the nerves of special sense are as readily affected as are those of common sensibility. The primary palor is supposed to be due to a very powerful constriction of the small blood vessels, but the anesthetic effect is not due to this, but to the direct action of the drug upon the nerve trunk.–(Wood.)

In the effort to secure a local anesthetic equally as efficient as cocain, but devoid of its extreme toxicity, pharmaceutical chemists have successfully produced on a commercial scale a number of synthetic preparations derived from the well known benzol group of compounds. Among the first of these was eucain, and a little latter a modification of this, less poisonous, was introduced. The first is now known as alpha eucane, and the latter as beta eucain. While these were improvements over the preparations from coca leaves, they lacked the desired solubility in water. Beta eucain is also presented as a lactate; the lactate is less toxic than its base. Beta eucain lactate is a white powder, possessing about the same anesthetic properties as cocain. It is considerably less toxic, is freely soluble in water, and its solution may be sterilized at a temperature of 115° C. without deterioration. This cannot be done with cocain. It is, however, an irritant of the tissues, sloughingly usually following its use. Its influence over the general system is so slight that fifteen grains may be injected without producing marked symptoms.

Tropacocain, or benzol-tropein, is a natural product obtained from the narrow leaves of the coca plant of Java, in a manner similar to cocain. It is an oily liquid, which solidifies in radiating crystals, and is soluble in chloroform, ether and benzine. Its local action is similar to that of cocain, without causing congestion of the mucous membrane with which it is brought in to contact. It is only half as toxic as cocain, but its injecting is apt to be followed by sloughing.

There are several other preparations, most of them proprietary, whose process of manufacture I have been unable to ascertain, but presume them to be chemical products. Their chemical formula is complicated, and their chemical name of interest only to experts in the higher branches of chemical science.

Nirvanin occurs in white, prismatic crystals, with a melting point of 185° C. It is readily soluble in water. It is a pronounced local anesthetic, but not equal to cocain. It is less toxic, a two per cent. solution may be freely used without deleterious effects. Eight grains may be given without producing toxic symptoms. It is said to be also an astiseptic, but its advantages over other preparations on the market are not sufficient to warrant its extensive use in dentistry.

Alypin is a white, crystalline powder, soluble in water and not decomposed by boiling. As a local anesthetic it is about as efficient as cocain, but possesses a much higher degree of toxicicity, and therefore it not suitable for dental use.

Stovine belongs to the the class of amino-alcohols, and was first produced before the French Academy of Medicine in 1904. It crystallizes in small brilliant scales, melts at a temperature of 175° C. and is very soluble in water. Its anesthetic action is greater than any of the other local anesthetics, and is much less toxic than cocain. It, however, causes intense hyperamia and sloughing.

Novocain occurs in colorless crystals. It is soluble in water and alcohol, and melts at a temperature of 150° C. It is the least toxic of all the local anesthetics. Its injection is followed by no swelling, and no hyperamia, the tissues remaining in a perfectly normal condition. As an anesthetic it is equal to cocain.

In conclusion, I invite attention to an instructive report of the Therapeutic Committee of the British Medical Association, published in *The British Journal of Dental Science*, April 15, 1909. The subject of their inquiry was to find a substitute for cocain in local anesthesia. They desire to find a drug with a lower degree of toxicity, in proportion to its anesthetic power, than cocain—a drug sufficiently soluble in water, whose solution should be stable, that is, would keep without deterioration, and one forming a solution capable of being sterilized by boiling. These properties they considered essential. They further required that it should cause no irritation, no injury to the tissues, and no deleterious after effects. Rapidity of absorption they did not consider especially desirable. If the drug is absorbed slowly it remains longer in contact with the nerve fibrils, and produces a more prolonged effect. They also considered it important to inject with the local anesthetic a local vaso-constrictor so as to restrict the anesthetic to the local territory, selecting for this adrenalin, and in their investigation gave prominent attention to the compatibility of adrenalin and the anesthetic. After an exhaustive investigation they reached the conclusion that of all the available local anesthetic agents novocain is the most satisfactory for general use. They report that its anesthetic action is equal to that of cocain, and its toxicity and general destructive powers on the tissues are very much less. Their ability, the thoroughness with which their work was done, and the fairness of their report entitle them to much credit, and their conclusions to the highest consideration of all dentists using local anesthesia in their practice.—*Dental Brief*, Sept 1909.

